# Peer Review for “Identification of COVID-19–Associated Hepatitis in Children as an Emerging Complication in the Wake of SARS-CoV-2 Infections: Ambispective Observational Study”

**DOI:** 10.2196/60168

**Published:** 2024-10-11

**Authors:** Petar Velikov

**Affiliations:** 1 Department for Infectious Diseases, Parasitology and Tropical Medicine, Medical University of Sofia, Sofia, Bulgaria

**Keywords:** COVID-19, coronavirus, SARS-CoV-2, liver, hepatic, hepatitis, child, children, pediatric, pediatrics, retrospective, observational, jaundice, youth, inflammatory, inflammation

*This is the peer-review report for “Identification of COVID-19–Associated Hepatitis in Children as an Emerging Complication in the Wake of SARS-CoV-2 Infections: Ambispective Observational Study.”*

## Round 1 Review

### General Comments

This paper [1] looks appropriate and relevant. Some corrections are needed. Additionally, none of the figures (except x-rays and study workflow) are properly visible on the PDF or Word version, so I cannot comment on their content. Please provide better-quality pictures, if possible.

Line numbers are not available, making the review and commenting much harder.

### Specific Comments

#### Major Comments

1. Correction suggested: COVID-19 infection as a term does not exist; it is a disease and has been incorrectly used by the general public and the media. Instead, SARS-CoV-2 infection should be used as this is the correct scientific term. This correction could be performed throughout the entire article, but it was specifically first found in the following sentence: “During active COVID-19 infection, milder disease phenotype was observed without necessitating hospitalizations whereas more severe patterns were seen in cases with MIS-C often associated with prolonged need for hospital admissions.”

2. Correction required in the following paragraph in the *Discussion* section: “With the emergence of newer VOC’S causing recurring waves of the pandemic, varied symptoms and post COVID-19 complications have been observed posing safety concerns even for the pediatric age group. In this scenario our study identified 37 cases with a unique presentation of acute hepatitis designated as CAH-C, whereas MIS-C could account for hepatitis in 10 cases amongst 15873 children screened in the district during the study period.” The number 15,873 in the *Results* section was previously mentioned as all screened patients regardless of age, and only 475 (2.99%) were actually children. Please correct or elaborate.

3. Is it not clear if informed consent was required or acquired for the follow-up phase?

4. A few more details about the adverse outcomes of the 3 children with multiple inflammatory syndrome in children (MIS-C) would be appreciated by the reader. Were there any relevant circumstances or peculiarities for these cases?

#### Minor Comments

5. References 7 and 8 might be wrong, as they do not relate to infections in children, as mentioned in the article. More appropriate references could be sought.

6. Sentence related to reference 17: Please correct to “at least 169” instead of “more than” to be exact with the terminology used in the original article. Otherwise, the meaning changes slightly.

7. Reference 18 does not exist, or the link no longer works, at least from my side.

8. Correction suggested: “On laboratory investigation: 35/37 CAH-C cases had RTPCR test negative for SARS- CoV-2 by the time when admitted.” “When admitted” should be changed to “the time of admission.”

9. A reference is missing for this sentence in the *Discussion* section: “The other concern is the association of biological false positivity (BFP) which might pose a diagnostic dilemma in cases of other infectious diseases, wherein similar febrile illnesses including dengue, chikungunya and enteric fever remain endemic in developing countries. Both these facts warrant further elaborate studies amongst larger populations and also during the vaccine trails among children.”

## Round 2 Review

No further comments. The authors have considered my suggestions. I recommend the article for publication.

## Abbreviations

MIS-C: multiple inflammatory syndrome in children
